# Supplementary material for: Factors determining cognitive, motor and language scores in low birth weight infants from North India
Source: PLoS One. 2021 May 12;16(5):e0251387. doi: 10.1371/journal.pone.0251387 (PMC8115769; doi:10.1371/journal.pone.0251387)
Supplement: S1 Table — (DOC) [file pone.0251387.s001.doc]

**S1 Table. Findings on univariable linear regression for cognitive, language and motor scores at 12 months of infant age in low birth weight infants from rural Haryana**

| **Variables** | **Cognitive Score** | | **Language score** | | **Motor score** | |
| --- | --- | --- | --- | --- | --- | --- |
|  | *Unstandardized ẞ-coefficient (95% CI)* | **P-value** | *Unstandardized ẞ-coefficient (95% CI)* | **P-value** | *Unstandardized ẞ-coefficient (95% CI)* | **P-value** |
| **HOUSEHOLD CHARACTERISTICS** | | | | | | |
| **Quintiles**  1 (Least poor)  2  3  4  5 (Poorest) | Ref  -3.31 (-6.46, -0.16)  -5.44 (-8.65, -2.25)  -4.80 (-7.99, -1.61)  -4.09 (-7.26, -0.93) | 0.040  0.001  0.003  0.011 | Ref  -2.57 (-4.94, -0.19)  -4.85 (-7.25, -2.84)  -5.86 (-8.27, -3.46)  -5.41 (-7.79, -3.03) | 0.034  <0.001  <0.001  <0.001 | Ref  -1.38 (-4.12, 1.43)  -1.05 (-3.90, 1.79)  -2.14 (-4.98, 0.70)  -2.77 (-5.58, 0.05) | 0.336  0.467  0.139  0.053 |
| **Religion**  Hindu  Muslim  Others^¶^ | Ref  -3.13 (-5.84, -0.42)  -0.12 (-11.78, 11.54) | 0.024  0.983 | Ref  -3.52 (-5.58, -1.46)  2.97 (-5.89, 11.84) | 0.001  0.509 | Ref  -3.56 (-5.92, -1.18)  5.72 (-4.49, 15.93) | 0.003  0.272 |
| **Social class^£^**  General  Other Backward Class (OBC)  Scheduled Caste/Tribe (SC/ST) | Ref  -1.96 (-4.67, 0.74)  -1.85 (-4.42, 0.71) | 0.153  0.156 | Ref  -2.43 (-4.49, -0.37)  -2.71 (-4.67, -0.76) | 0.021  0.007 | Ref  -1.62 (-4.00, 0.76)  -0.36 (-2.62, 1.89) | 0.183  0.752 |
| **Type of family**  Nuclear  Joint | Ref  3.82 (1.51, 6.14) | 0.001 | Ref  2.13 (0.35, 3.91) | 0.019 | Ref  1.58 (-0.47, 3.63) | 0.131 |
| **MATERNAL AND PATERNAL CHARACTERISTICS** | | | | | | |
| Maternal age (years) | -0.53 (-0.80, -0.27) | <0.001 | -0.34 (-0.55, -0.13) | 0.001 | -0.34 (-0.58, -0.11) | 0.005 |
| Maternal years of education | 0.51 (0.30, 0.71) | <0.001 | 0.53 (0.38, 0.69) | <0.001 | 0.39 (0.21, 0.59) | <0.001 |
| Father’s age (years) | -0.37 (-0.59, -0.16) | 0.001 | -0.29 (-0.45, -0.13) | 0.001 | -0.24 (-0.43, -0.05) | 0.014 |
| Father’s years of education | 0.36 (0.14, 0.58) | 0.001 | 0.37 (0.21, 0.54) | <0.001 | 0.27 (0.08, 0.47) | 0.006 |
| **Father’s occupation**  Employed in government/private firm  Daily wage earner  Self-employed (own business/farming)  Unemployed | Ref  -3.44 (-6.12, -0.76)  -1.19 (-3.60, 1.21)  1.36 (-3.83, 6.55) | 0.012  0.329  0.607 | Ref  -2.75 (-4.79, -0.69)  -0.49 (-2.33, 1.35)  -0.46 (-4.43, 3.51) | 0.009  0.601  0.821 | Ref  -2.28 (-4.64, 0.09)  -1.13 (-3.26, 0.99)  -2.04 (-6.62, 2.55) | 0.059  0.295  0.383 |
| **BIRTH RELATED CHARACTERISTICS** | | | | | | |
| **Place of delivery**  Home  Government facility  Private facility | Ref  1.62 (-0.75, 3.99)  4.43 (1.45, 7.41) | 0.180  0.004 | Ref  1.84 (0.04, 3.63)  5.14 (2.89, 7.39) | 0.045  <0.001 | Ref  0.42 (-1.68, 2.53)  1.47 (-1.17, 4.11) | 0.694  0.273 |
| **Type of delivery**  Normal  Caesarean section | Ref  -2.10 (-12.57, 8.36) | 0.693 | Ref  4.71 (-3.29, 12.71) | 0.248 | Ref  5.85 (-3.36, 15.05) | 0.213 |
| **Birth order**  1  2-3  ≥4 | Ref  -3.56 (-5.77, -1.34)  -8.03 (-10.89, -5.17) | 0.002  <0.001 | Ref  -3.60 (-5.28, -1.93)  -6.82 (-8.99, -4.64) | <0.001  <0.001 | Ref  -2.27 (-4.25, -0.29)  -4.52 (-7.09, -1.95) | 0.025  0.001 |
| **Parity**  Multiparous  Primiparous | Ref  4.84 (2.76, 6.92) | <0.001 | Ref  4.52 (2.95, 6.10) | <0.001 | Ref  2.91 (1.06, 4.76) | 0.002 |
| **INFANT CHARACTERISTICS** | | | | | | |
| **Sex of the baby**  Male  Female | Ref  0.35 (-1.74, 2.44) | 0.743 | Ref  -0.23 (-1.83, 1.37) | 0.780 | Ref  2.28 (0.46, 4.12) | 0.014 |
| Birth weight (grams) | 0.004 (-0.002, 0.009) | 0.238 | 0.003 (-0.002, 0.008) | 0.236 | 0.005 (-0.0002, 0.01) | 0.057 |
| Gestational age (weeks) | 0.02 (-0.49, 0.53) | 0.928 | -0.15 (-0.55, 0.24) | 0.443 | -0.14 (-0.58, 0.31) | 0.551 |
| Early initiation of breastfeeding | -0.15 (-2.26, 1.97) | 0.891 | 0.25 (-1.38, 1.86) | 0.766 | 0.19 (-1.67, 2.06) | 0.836 |
| Exclusive breastfeeding at 3 months | 1.72 (-0.38, 3.82) | 0.108 | 1.28 (-0.33, 2.87) | 0.118 | 1.74 (-0.09, 3.57) | 0.063 |
| HAZ at 12 months | 3.39 (2.46, 4.33) | <0.001 | 2.64 (1.92, 3.36) | <0.001 | 3.28 (2.47, 4.09) | <0.001 |
| WHZ at 12 months | 2.26(1.18, 3.34) | <0.001 | 2.37 (1.55, 3.19) | <0.001 | 2.36 (1.42, 3.29) | <0.001 |
| **HOME ENVIRONMENT** | | | | | | |
| PROCESS Score at 12 months | 0.28 (0.22, 0.34) | <0.001 | 0.26 (0.22,0.30) | <0.001 | 0.19 (0.14, 0.24) | <0.001 |
| **MORBIDITY** | | | | | | |
| No. of episodes of “Diarrhoea”  No. of episodes of “Pneumonia”  Any hospitalization during infancy | -3.76 (-5.38, -2.14)  -0.64 (-2.66, 1.39)  -0.43 (-2.48, 1.62) | <0.001  0.538  0.679 | -3.11 (-4.35, -1.87)  -0.34 (-1.89, 1.21)  -0.24 (-1.81, 1.33) | <0.001  0.664  0.762 | -3.52 (-4.94, -2.01)  -1.50 (-3.26, 0.27)  -1.42 (-3.22, 0.38) | <0.001  0.095  0.122 |
| **MATERNAL AND INFANT PSYCHOSOCIAL CHARACTERISTICS** | | | | | | |
| PHQ-9 Scores at 6 weeks of infant age | 0.10 (-0.26, 0.46) | 0.589 | -0.02 (-0.30, 0.26) | 0.899 | -0.02 (-0.34, 0.30) | 0.901 |
| Mother-infant bonding at 6 weeks | -0.10 (-0.29, 0.09) | 0.294 | 0.06 (-0.09, 0.20) | 0.429 | -0.09 (-0.27, 0.07) | 0.238 |
| Maternal self-efficacy at 12 months | 0.67 (0.30, 1.05) | <0.001 | 0.85 (0.57, 1.13) | <0.001 | 0.59 (0.26, 0.91) | <0.001 |
| Infant temperament scores at 12 months | -0.11 (-0.18, -0.04) | 0.001 | -0.09 (-0.14, -0.04) | <0.001 | -0.12 (-0.18, -0.06) | <0.001 |
| **INTERVENTION** | | | | | | |
| Kangaroo Mother Care (KMC) | 0.21 (-1.84, 2.26) | 0.838 | -0.90 (-2.47, 0.67) | 0.259 | -0.85 (-2.65, 0.96) | 0.356 |
