# Supplementary material for: Factors determining cognitive, motor and language scores in low birth weight infants from North India
Source: PLoS One. 2021 May 12;16(5):e0251387. doi: 10.1371/journal.pone.0251387 (PMC8115769; doi:10.1371/journal.pone.0251387)
Supplement: Attachment — Submitted filename: Response to reviewer.doc [file pone.0251387.s002.doc]

**Response to reviewer’s comments**

**Reviewer 1**

1. One aspect that the authors should point out more clearly is that the sample they investigate is only of slightly low birth weight. It would be interesting to extend the study with a sample of very or extremely low birth weight children. On the other hand, it would have been desirable to compare the results of the sample studied to a control group of children with normal birth weight.

**Response:** We agree with the reviewer on this point. We have added in the limitation the following sentence- “One key limitation is that the study subjects for this analysis were largely stable late preterm or term infants small for gestational age and were only slightly low birth weight. Therefore, the findings may only be applicable to this specific subset of infants”.

We also agree with the reviewer that it would have been desirable to compare the results of the sample studied to a control group of children with normal birth weight but as this was a secondary analysis of data from a trial involving only low birth weight infants, such a comparison could not be drawn.

**Reviewer 2**

1. Please include some numerical results in the 'results' section, e.g. where there was evidence of association between an exposure and outcome, the magnitude of the estimated difference or ratio with its 95% confidence intervals and p-value.

**Response**: We have done the suggested change in the abstract

2. Introduction
   - the last sentence on line 78 best belongs in the methods; indeed it is already stated in the first paragraph from line 83, so should probably be removed from here.

**Response:** We thank the reviewer for this suggestion. We have removed the sentence from the introduction section.

3. Methods
   - line 91: a better title would be 'participants and data collection'. It would be helpful to add to this section how the size of the primary trial was determined.

**Response:** We have done the suggested change in the title and also added the section of sample size calculation for the primary trial.

4. Line 115: a better title would be 'outcomes'

**Response:** We have done the suggested change in the title

5. Line 126: a better title would be 'exposures and other variables'. These suggested titles are similar to the sub-sections of the reporting guidelines. Please ensure, having used these titles, that the information reported under each section matches the titles, and move around the ones that don't.

**Response:** We have done the suggested change in the title and also checked for the content under each of these sections.

6. Statistical analysis
   - it would be better if the authors presented a brief summary of the potential conceptual relationships between the variables explored for adjustment and (1) the outcome (2) the main exposures, rather than wholly relying on a purely data-driven variable selection approach such as described in line 160. In this summary, the authors should consider the potential directions of the associations between the variables and each of the outcome and main exposure. The reason being that adjustment should only be undertaken for variables that meet the authors' criteria for independent association with the outcome and with the main exposure (e.g. those described in lines 165-167) and not on the causal pathway between the exposure and outcome.

**Response:** We adopted a predictive approach rather than a risk factor approach i.e., we did not aim to document the association between a particular risk factor with the cognitive outcomes. Therefore, we did not consider potential conceptual relationships between the variables explored for adjustment, probably on lines similar to Directed Acyclic Graphs (DAG). We however looked for variance inflation factor (Vif) in order to quantify the extent of correlation between predictor variables in a model.

7. Given the shapes of the associations between relative cognitive scores and relative composite language scores with LAZ scores, WHZ scores, PROCESS scores, birth order, it may be worth exploring any departures from linearity in these associations, e.g., by testing whether quadratic terms for these exposures (in addition to the linear term) are significant in the 'final' models, and if so, perhaps incorporate these terms early on in the adjusted model building process.

**Response:** Our GAM plots indicate that most of the described associations are approximately linear which justify our including them as linear terms in the regression models. However, because the association between weight for height z-scores (WHZ) and motor function scores seems to deviate from linearity, we have included the following sentence in the discussion:

“It should be noted from Figure 3 that the association between WHZ and composite motor scores may not be linear. Therefore, it would have been more appropriate to include WHZ as quadratic or logarithmic or exponential term or in other transformed form to achieve linearity for regression analysis. However, because such terms are difficult to interpret and communicate, we chose to include it as linear term in addition to showing the shape of association in figure 3.

8. It is generally more appropriate to report likelihood ratio or other global p-values for categorical variables with more than two levels, such as birth order, than the Wald test p-value for each level. I'm also wondering whether it might have been better to fit birth order as an ordinal explanatory variable, i.e., estimate the effect per unit increase rather than categorise it; did the authors explore this (if so, how)?

**Response:** We thank the reviewer for this suggestion. We have now reported the P-value based on the likelihood ratio test. We have also added a statement regarding this in the footnote of table 2. We did the suggested analysis by including the birth order as a continuous variable and the findings have been presented in the results section. The inclusion of birth order as a continuous variable in the model did not affect the associations for other variables with the cognitive and language composite scores.
